# Epidemiological geomatics in evaluation of mine risk education in Afghanistan: introducing population weighted raster maps

**DOI:** 10.1186/1476-072X-5-1

**Published:** 2006-01-03

**Authors:** Neil Andersson, Steven Mitchell

**Affiliations:** 1 Centro de Investigación de Enfermedades Tropicales (CIET), Universidad Autónoma de Guerrero, Acapulco, Mexico; 2 CIETcanada, Ottawa, Ontario, Canada

## Abstract

Evaluation of mine risk education in Afghanistan used population weighted raster maps as an evaluation tool to assess mine education performance, coverage and costs. A stratified last-stage random cluster sample produced representative data on mine risk and exposure to education. Clusters were weighted by the population they represented, rather than the land area. A "friction surface" hooked the population weight into interpolation of cluster-specific indicators. The resulting population weighted raster contours offer a model of the population effects of landmine risks and risk education. Five indicator levels ordered the evidence from simple description of the population-weighted indicators (level 0), through risk analysis (levels 1–3) to modelling programme investment and local variations (level 4). Using graphic overlay techniques, it was possible to metamorphose the map, portraying the prediction of what might happen over time, based on the causality models developed in the epidemiological analysis. Based on a lattice of local site-specific predictions, each cluster being a small universe, the "average" prediction was immediately interpretable without losing the spatial complexity.

## Background

Colourful and seductive, geographic information systems (GIS) offers status symbol technology that researchers and development agencies rush out to purchase. The imagery conjured up by a map is immediate and compelling: if we believe the map, we seem to know where the problems are and, it should follow, where interventions should take place to address them. In many aspects of public sector planning, GIS can help information sharing. Analysis of the spatial dimensions of public service programmes can also enhance coordination and specificity of action to the local conditions [1]. The hope that GIS might increase programme efficiency and investment choices, however, remains largely unfulfilled. This depends on the elusive integration of GIS with formal analysis of risk and resilience.

We developed the term epidemiological geomatics for an approach reaching beyond visual overlays of spatial data, to include modelled interactions between layers that inform population based predictive models. Using this approach, planners who need to know more than just the locality of a particular problem can visualise epidemiological models of programme performance.

Several problems obstruct the use of maps in epidemiology. The inaccessible "black box" nature of GIS technology has led some to believe that maps might solve a problem they cannot solve. Like any presentation tool, the information that maps convey can be no better than the data that goes into them. The attractiveness of the imagery is far from a guarantee that the evidence has meaning. Meta-data of maps – where and how the evidence was collected – are often taken for granted, yet they can change completely the interpretation.

A map showing the location of a place with a high rate of, say, a particular disease, is a very partial model of reality. Most maps that show disease patterns cover the geographic domain where the evidence is generated. A case in point is the National Center for Health Statistics (NCHS) Atlas of United States Mortality [2] that portrays the national rates of various conditions. The cases in County X are assigned to a polygon demarcated by health service areas, where it is related to the population to produce a rate. The rate is colour coded and the resulting image offers the planner an *average *indicator level across the polygon. It has a meaning only insofar as the average has a meaning. But averages miss the extremes, often the most vulnerable who are the focus of much public health planning. Averages are notoriously distorted by outliers, without fully reflecting those outliers. Sometimes it is the outlier that most merit the public health action.

One might also find a health service area or district with a low mortality or coverage immediately adjacent to one with a very high level of the same indicator. The "boundary" between two polygons can be an unrealistic and abrupt change. Since it does not reflect any gradient between highest and lowest rates, the map loses coherence and, with that, much of its use in planning.

Almost all countries have a routine data system that makes some attempt at collecting cases or documenting service activities. These can sometimes be useful for mapping. The challenge intensifies with data from sample surveys. In order to fill in a polygon based on a sample, the survey must be of a sufficient sample size and it must be chosen in such a way as to permit generalisation to the whole polygon. This must be done for each polygon.

Then there is the difficulty of compatibility of data sets. Data come in different formats and they refer to different domains, at different times. In the Afghanistan case, a mine survey mapped the physical location of the mine fields. Existing services were catalogued and mapped, and these data can be related to where the population is thought to live. But in Afghanistan the last census was done several decades ago. Even the 1990 "update" was altered drastically by several new phases of the war. Unrelated sample surveys with varying objectives provide data from some places but not from others. This information asymmetry is typical of situations where maps are needed most. Even outside of emergency situations, data on environmental risks, population distribution and services are rarely coterminous.

## Case study from Afghanistan

Land mines are an intrinsically spatial issue – land mines alter geographic access – and integration of epidemiological risk analysis with GIS should offer substantial benefits for planning and evaluation of mine action. The harsh conditions of Afghanistan offered an opportunity to test the added value of GIS in epidemiological evaluation[3]. Even in the mid-1990s, Afghanistan was not without existing data. Because of the strategic importance of the country and its particular history in the last years of the Cold War, its geography was unusually well documented. For years, US spy satellites followed movement of people, documenting every contour and cave. When the USA pulled out of the region, they made available huge banks of data accessible to the humanitarian organisations working in Afghanistan.

The quality and comprehensiveness of the topographic data were complemented, if not entirely matched, by moderately up-to-date mapping of landmines. The survey of mined areas offered some understanding of the distribution and density of landmines or unexploded ordnance (UXO) contamination. With all this wealth of spatial information, the missing elements were the people and their behaviour. Sample surveys of the impact of landmines and the evaluation of mine risk education have contributed elements towards a comprehensive geographic information base on mines and mine action in the country.

But these sources of evidence, each rich as it may be, were uneven, fragmented and not integrated in a usable way for decision-taking. Mine risk education in Afghanistan, prior to a CIET evaluation conducted in 1997/98 [4], rested on two main types of education. Direct training through lectures at community level was conducted by three non governmental organisations; they were supposed to establish "mine committees" in each community where training had occurred, to sustain and to continue the risk education. The second mine risk education modality was through a soap opera, called *New Home New Life*, on the BBC's Afghanistan Service.

The CIET evaluation found many people who did not even know they were listed as "trained" in the direct training scheme. There was some evidence of a positive impact of direct training in the mine-affected areas, for example, a higher likelihood of reporting a mine or UXO, but not all results from the training programme were positive. Those who said they received direct training were more likely to declare high risk attitudes and practices (such as searching for scrap metal and considering someone brave who goes into a mined area but is not a deminer). Among trainees, there was a significantly higher risk of injury after the training programme began. They suffered higher risk of injury affecting the upper part of the body (hands, arms, eyes and torso); injuries most likely resulting from attempts to diffuse the mines rather than mines being stepped upon. This suggests inappropriately heightened confidence in handling mines among those who received training. One explanation for these findings was that much of the mine risk training was conducted by deminers. They may have taught what they know about mines – for example, where the detonator is housed and how it works – thus engendering the false feeling of security.

The BBC radio soap-opera *New Home New Life*, had a measurably positive effect on reinforcing appropriate mine behaviour, frequency and type of injury.

## Epidemiology behind maps, and using maps in epidemiology

### The planner's question

The fact that an action is planned and paid for does not mean it is implemented. The fact that a service is offered does not mean people make use of it. And, if they do take up services that are offered, it does not mean these services have the desired effect. Like almost any public sector intervention, mine risk education does not work equally well everywhere and, in some places, it does not work at all. This is the issue at the heart of evidence-based planning: what *actually happens *is what counts, not only what is intended to happen.

The central exercise in evidence-based planning is to quantify the gap between what is intended and what actually happens, and to identify the particular mix of circumstances under which an intervention is effective. Planners need to know the coverage achieved by each service, but they also need to know what allocation of resources it will take to close the gap between intended impact and actual impact. More than the precise identity of the localities where services work or do not work, planners need to know about the particular mix of circumstances under which an intervention works.

### A framework for epidemiological study

The cross design of techniques known as the CIETmethod [5] – also known as sentinel community surveillance (SCS) [6] or service delivery surveys (SDS) [7] – tries to maintain epidemiological coherence of evidence introduced into planning. The method relies on a panel of clusters weighted to link the sample to the universe it represents.

Cyclical contacts with these a representative panel of clusters (Table 1) involve a concentration of measurement resources in time and place, an intense focus of quantitative and qualitative methods in a panel of mini universes. The ability to repeat measurement in the same place makes impact estimation relatively straightforward. These households can be contacted in successive cycles, perhaps a year or two years later, to measure differences over the period. These differences can be related to programmatic input and other factors that might be vary across different clusters. The impact assessment is based on the time sequence and the heterogeneity between clusters.

**Table 1:** The CIET fact-finding/feedback cycle

1. Identification of the issue to be researched, for example, access to water and sanitation, access to and use of services, adequate food supply, etc.
2. Ordering and analysis of data from routine sources and previous studies, attempting to align data in three analytical categories: impact, coverage, and costs.
3. Development of the instruments including precise objectives, questionnaire, key informant interviews, data entry format, and report outline.
4. Pilot testing including data entry and analysis.
5. Fieldwork including household questionnaires and qualitative techniques (key informants, observation, focus group discussions).
6. Data entry and preliminary analysis, identification of confounders and effect modifiers.
7. Feedback and interaction in sentinel communities for interpretation and strategy development.
8. Completion of analysis, refinement of programme options.
9. Development of the communication strategy that can be consultative process in the same clusters.
10. Communication of results to all communities, development of strategies of action to resolve issues.

The contiguous households in each cluster to permit the analysis of local factors *in the context of household-level occurrences*. Some environmental factors might be quantified easily (for example, presence of school, cost of drugs) or they may be more qualitative (adequacy of sanitation, level of participation in community affairs). If these factors affect the whole cluster, comparisons can be made between clusters or groups of clusters.

Costs of public services (and costs of not accessing public services) can vary from place to place in such a way that a particular solution may have a favourable cost-effectiveness ratio in one place but not in another. Remoteness can drive up transport costs or create monopoly conditions for service providers. Household or cluster-specific cost data can be collected from key informants and fed into the mix. Decisions on resource allocation can thus become more flexible, adapting to local cost realities.

### A model with predictive value

The task of epidemiological geomatics in Afghanistan was to predict what might happen with mine education based on the empirical evidence in those clusters.

We are used to hearing a statistic with predictive value derived from a sample survey. For example, an opinion poll about what people think prior to an election can have predictive value regarding an election outcome. A service delivery survey may attempt to establish the coverage of a given public service, and to gain insight into why the services are not utilised optimally.

Sampling, so familiar in epidemiology, has yet to find a comfortable fit with GIS. Formal steps can link a sample with a given domain. In Afghanistan, we stratified the country into the four UN operational regions to ensure the sample was balanced between those domains. Second stage random selection of districts in each UN operational region produced a sample of 48 districts. We stratified each sample district by population density, urban and rural, to ensure appropriate balance in population proportion in these substrata. From a list of all candidate communities in each substratum, giving each cluster a specified chance to enter the sample, we randomly selected survey communities. From a map of each survey community, we randomly selected a cluster of households. We then calculated the population weight for each fixed-size cluster (100–120 households), based on the population it was supposed to represent in the four UN regions.

The resulting sample of 86 sentinel clusters represented the spread and proportions of different conditions under evaluation. No sub-sampling was done within each cluster or sentinel site (100–120 contiguous households, each including 500–1000 people). This large and relatively fixed cluster size derives from an epidemiological concern to have sufficient data in each place for that "point" in the model to be interpretable. In effect, each cluster is a mini universe, and the sample of clusters is a lattice of mini universes.

### Weighted clusters

The unit of mapping is usually physical space – square kilometres. Planners are often concerned about physical space but they are usually even more concerned about the people who live and work there. We "weight" the clusters to relate them to the population, not the land area. Some segments of a country will be densely populated, and others sparsely populated. It is possible to introduce this population dimension by applying the sample weights to link the model to the "country", with the weight of each cluster proportional to the population it represents.

**Figure 1:**
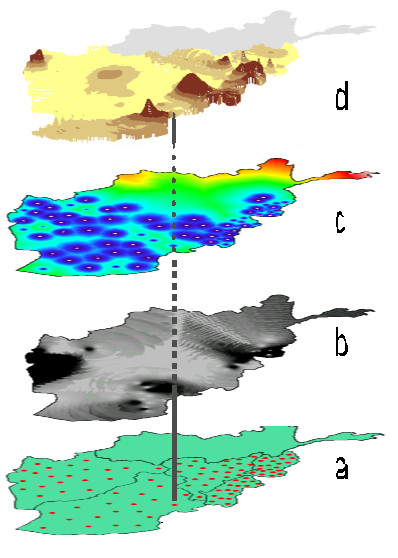
**Layers in a population-weighted raster, based on a cluster sample**. a) position of sentinel sites or "tent-poles", b) population weights, c) friction layers and d) raster drape (top) where the shade is set by the height of the tent-pole and the extension of an interpolation based on the population weights.

This involved building a "friction surface" under the map, rather like the underlay of a carpet, with circles radiating outwards around each one of the 86 sentinel clusters (Figure 1, the lowest segment represents the location of the "tent-poles", the second layer from the bottom is the interpolated weight layer, and the third layer is the friction surface). The friction surface for a large and small town would look a bit like a big stone and a small stone dropped some distance apart into a calm pool. The ripples caused by the big stone go further and have more influence on the overall picture than the ripples from the small stone. This friction surface, set to match the population weight of the cluster in the overall sample, sets the influence each point has on the overall map that they make up together.

**Figure 2:**
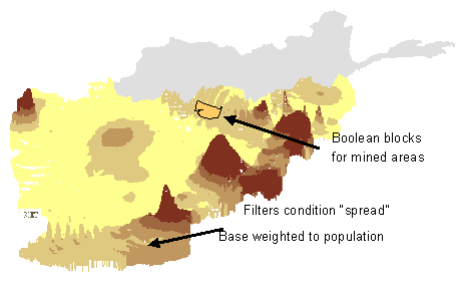
**"Ortho" or three-dimensional view of population-weighted raster**. Distribution of households that say land mines affect their livelihood (Afghanistan 1997).

The friction surface to introduce population weights was initially created using the *Cost *function in Idrisi. The friction surface is an inverse of the interpolated cluster weights: where clusters were under-represented in the sample, having a higher population weight, friction levels during interpolation were lower. Therefore under-represented clusters were allowed to "spread out further" on the surface during the interpolation (acceleration). Conversely, over-represented clusters moved higher frictions during interpolation and their overall effect thus reduced.

Population weighting transforms entirely the use of maps for planning. The clusters in our Afghan sample were each referenced to the population they represent by a weight. On the map, this weight determined the spread of a colour or shade *in proportion to the population*. The net result is that the map can show 40% of the country in a given shade of green, when 40% of the people who live in that country are affected by the exposure represented by that shade. This makes immediate sense for planners, who usually think about populations rather than square kilometres.

The practical jump was to apply the facts from the mini universe that was each cluster to the remainder of the sample.

### Raster modelling

Most public sector planners have at least heard of vector and raster mapping systems. Perhaps because of the long history of the use of vector mapping in physical planning, where exact locations of roads, sewers or archeological remains are points and lines, vector-based packages (like ArcView and MapInfo) are well known. A characteristic of vector maps is that the exact location of every house in a given cluster or sample can be identified in a database. In some applications, this can be useful, allowing "drill-down" to show the street number of the house where a situation X is located. Usually these are more data about occurrence – and less about the relationships around occurrence – than planners need. Planners need to know more about sets of circumstances associated with impact. They need occurrence data on the convergence of several different factors, rather than exquisite accuracy on any single occurrence.

The ethics of holding highly identifiable data like this must be questioned. Confidentiality is a well-known complication of a drill-down ability, especially in this age of remote access to information. There are detailed research protocols that govern this from a purely ethical standpoint, but having intensely personal details on the identities and locations of cases or exposures does pull in the opposite direction to transparency and participation in planning. Data cannot be made widely accessible, which defeats some of the exercise of bringing the maps into the planning process – to increase the stake holding in the evidence.

Sample size of a cluster sample is a particular issue in vector mapping. For each polygon, sufficient clusters are needed to provide a reasonable representation of the domain. Aggregated over a country with several hundred districts, this means the survey must include a huge number of clusters (or individual households).

A raster-based or fusion raster-vector GIS package (like Idrisi or CIETmap) essentially creates surfaces that "drape" over a lattice of identified places from where the data are available. This fits comfortably with a cluster survey: clusters can be likened to a lattice of "tent-poles", each pole potentially of a different height, depending on the value of the indicator. Draping a tent over the grid of tent poles produces a surface that shows the different pole heights. This surface or raster offers an opportunity to assign different colours to different areas of the canvas, depending on the height of the pole holding up each portion. A high pole, an elevated indicator, might have a dark tone to indicate a high level of coverage with a given mine action. A pale shade might illustrate a low level of coverage.

**Figure 3:**
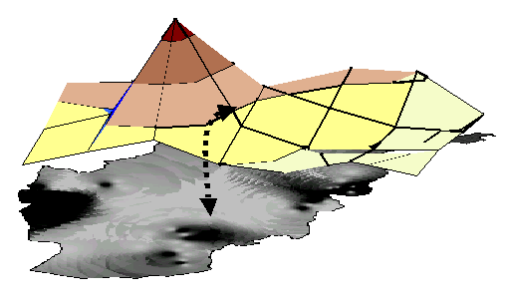
**Schematic portrayal of a raster being laid over a weight layer**. The interpolation of the colour change is based on the population weight.

Figure 3, a schematic representation of two rasters or surfaces, depicts a high pole, representing a high level of an indicator, and a low pole. Somewhere between these two poles, at a point determined by the relative importance of each in the population (the friction surface), the colour changes from dark to lighter.

This interpolation that sets the exact point of colour change between two clusters is done by the mapping package, but the operator should have a say in how it happens. The raster maps generated for the Afghanistan mine awareness evaluation originally used Idrisi with global inverse-distance weighting (IDW). The friction layer was then used to adjust the interpolations to take into account the population weights. In CIETmap, an open source epidemiological geomatics software, an adjusted IDW formula takes into account population weights during interpolation. Users can control the influence of distance and population weights on the resulting image.

The friction surface generated in Idrisi using the *Cost *module creates an isotropic effect around each cluster. This is a limitation of these early population weighted maps as, in reality, population patterns would not be symmetrical in this way. A non-isotropic function, such as Idrisi's *Varcost*, allows for effects to vary in different directions if sufficient data on population movement, roads and physical boundaries (rivers, lakes or forests) are added. In the Afghan case, regional inferences were more important than specific local inferences and, consequently, the cruder picture generated by the *Cost *module was probably sufficient for the purpose.

Raster-based modelling also allows several surfaces to interact, which is where epidemiological causality analysis comes into the picture. One type of occurrence data from a cluster can be set in a formula that interacts it with another datum for the same cluster. For example, Figure 3 shows a schematic depiction of one surface being draped over an existing surface. This is what happens when an "intervention" coverage layer is added to an "outcome" layer, for example, the effect on behaviour of BBC listenership. The resulting new layer, the modified outcome, is based on the interaction at each cluster; the effect of listening to the BBC on the behaviour *is *the actual cluster-specific effect, after taking account of other possible influences (see below).

**Figure 4:**
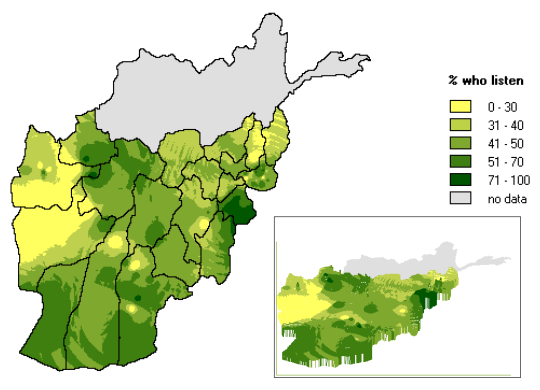
**Raster map showing Level 0 indicator**. Population-weighted distribution of listenership to BBC soap opera New Home New Life including ortho (three-dimensional) view.

The surfaces interact cluster-to-cluster, based on real data from each cluster. We can also allow for the other factors we know, like literacy and food security, that also influence how the social costs of land mines will be felt and how the mine action will be received differently in each cluster. As each subsequent layer is "lowered" onto the existing surface, the cluster-specific interaction between the layers sets the new contours of the occurrence relations (Figure 4).

Although the household data from each cluster might be exquisitely accurate for that place, raster modelling extrapolates this finding radially outwards. As the result from each cluster radiates outward, it "seeks" and interpolates with every other result radiating out from each other cluster.

It may eventually be possible to condition the outward radiation, with a function like Idrisi's *Varcost*. Thus, one might show the influence of physical or cultural barriers. However, the model does locate the proportion of the population represented by the cluster as geographically contiguous to the cluster. In reality, this may not be the case and so the interpretation for planning is "in places like this", rather than "in this specific place". Based on a reliable sample, stratified by region and urban/rural character as in Afghanistan, this allows the planner to make strategic choices and to focus resources accordingly.

### Levels of indicators and epidemiological analysis for planning

Different levels of indicators summarise what planners need to know, progressing from simple description, through analysis of causality to modelling programme investment. A basic indicator gives a bland description of occurrence, the corollary of an opinion prevalence in an opinion poll (Level 0 in our nomenclature, Table 2). This can be the proportion of the population directly affected by a problem, in the landmine context those who have lost a hand or a leg, or it can be the proportion who have been trained in mine risk education or who listened to the BBC soap opera. Overlays of Level 0 coverage indicators permit identification of low cover areas and high risk areas. To a very limited extent, Level 0 indicators permit coarse interpolation of where there is convergence of coverage and effect and, therefore, a first indication of where to focus resources to make up the shortfall. The problem of inappropriate inferences from this sort of data has been referred to as the "ecological fallacy" [8].

**Table 2:** Five levels of indicators for planning

Level 0: Descriptive frequencies (percentages, rates) and characteristics (averages, modal values) provide an overview of the occurrence a given risk-taking behaviour, or the coverage of a given health programme.
Level 1: Individual risk estimates (unbiased odds ratios) reflect the average risk of an individual "exposed" to a given mine awareness programme, in comparison with the average unexposed individual.
Level 2: The expected gains are the number of cases that can be "saved" – after taking into account all the other factors that could explain the association – by a given intervention.
Level 3: Combinations of programmes can produce additive or multiplicative gains, which are enormously important in estimating the cost options for planners.
Level 4: Since is it rarely possible to invest fully in all programme options simultaneously, this models programme structure based on *partial investment*. It also anticipates partial uptake of the programme and local cost variability

The Level 1 indicator avoids this problem by combining exposure and outcome, to estimate the *average individual risk *(odds ratio) of inappropriate behaviour of an individual who had received mine risk education. In these higher level indicators, the concern is causality: does the exposure decrease the risk of mine accidents?

Table 3 shows the evidence of impact of the BBC programme, reflected in terms of number of cases before and after programme commencement in late 1994, in response to an earlier study on social costs of land mines [9]. The 1998 data refer to the type of injury sustained and mine-smart behaviour in fixed settlements only (excluding nomads and camps). Although it is not clear why listeners had higher risks than non listeners prior to 1994, the evidence suggests that listening in some way neutralises part of the huge increase in risk associated with the post-1994 dramatic increase in population movement.

**Table 3:** Evidence of impact of *New Home New Life *on the number of mine/UXO events before and after 1994, upper limb (tampering) injuries after 1994 and inappropriate attitudes and practices

	Listen to *New Home New Life*	
	
	yes	no
Mine/UXO events 1. Cases 1979–93 (per 10000 person-years)	21 (6.802)	9 (1.898)
2. Cases 1994–97 (per 10000 person years) Risk of a case occurring after 1994: OR 0.28, 95%CI 0.07–0.99 (in urban mined areas)	9 (14.577)	14 (14.763)
3. Upper limb injuries as proportion of all injuries among survivors of mine accidents after 1994 Risk of an upper limb injury after 1994: OR 0.12, 95%CI 0.02–0.82 (in urban areas)	3/14 (21%)	11/16 (69%)
Inappropriate attitudes and practices (search for metal, consider non-qualified person brave if go into mined area)		
4. With easy radio access (%)	377/4207 (9%)	65/423 (15.4%)
5. Difficult radio access (%) Risk of inappropriate behaviour, stratified by ease of access to radios OR 0.61, 95%CI 0.44–0.84	2/83 (2.4%)	378/4216 (9%)

In addition to the protection against mine events, there was also evidence that listening to the BBC affected mine-smart attitudes and practices. A BBC listener was only one half as likely to support risky behaviour or attitudes compared with a non listener (among those with easy access to a radio 377/4207 compared with 65/427; among those with difficult access to a radio, 2/83 compared with 311/4410; unbiased estimate OR 0.52 95%CI 0.39–0.69, unbiased RD 5.4% 95%CI 3.1–7.7). There is notable confounding by ease of listenership: those who have least access to radios also have different pressure, probably economic, to engage in risky behaviour. To be sure that the association was not explained by chance or one of the many other factors that could be linked with the risk education, the Mantel-Haenszel procedure was applied sequentially for each of the potential explanations [10]. The final model was confirmed using logistic regression analysis [11].

In the Afghanistan project, there were more than 70 of these factors to be excluded as possible explanations. None of these could explain the apparently protective effect of the BBC's *New Home New Life *programme. In contrast with the BBC programme, no such protective effect could be found for the direct training programmes. Such evidence as could be obtained pointed to significantly more upper limb injuries after the direct training, compatible with inappropriate tampering with the devices. Direct training was also associated with more inappropriate attitudes and practices (odd ratio 1.73, 95%CI 1.48–2.02; 329/2917 in the programme areas reported these attitudes and practices, compared with 426/6207 in the non programme areas). This association could not be explained by any of the possible confounders taken into account in the analysis.

The implication is that stopping or modifying the direct training programme could be expected to have a measurable reduction in upper body injuries and a decrease in inappropriate attitudes and practices (Table 4). When the effect of stopping or changing the direct training is combined with the BBC programme, there is an expected protective effect, greater than that of changing the training on its own and of the BBC on its own.

**Table 4:** Expected gains table (reduction in inappropriate behaviour) of BBC listenership and direct training

Actions	Odds Ratio (average individual risk)	Risk difference	Proportion requiring intervention	Gain**/1000 households
Universal listenership to the BBC *New Home New Life *soap opera*	0.61	0.046	53% current non-listeners	24.3
Stop or change current direct training programme*	0.57	0.044	32% in communities now exposed	14.1
Universal listenership and change direct training	0.52	0.066	67% is either non-listener or is exposed to training	44.2

*each excluding the effect of the others and the confounding effect of 'easy access to radios'

** 'Gain' is the risk difference divided by the proportion requiring intervention

Level 2 indicators or "expected gains" are based on the same epidemiological approach to exclude other possible explanations for the association. Derived from the risk difference (the incidence in the exposed minus the incidence in the unexposed), the expected gain is the central parameter for the public heath planner. The gain, simply put, is the number of cases that can be "saved" – after taking into account all the other factors that could explain the association – by a given public health intervention. The gain is the risk difference divided by the "proportion requiring intervention", the proportion of the population exposed to a risk, or the proportion without coverage in the case of a positive intervention. The expected gain has proved useful in assessing the planning importance of different interventions. Table 4 shows a completed gains table for the case of direct training and BBC listenership influencing mine risk education.

Level 3 indicators model the combination of actions; what, for example, would happen if the training was made more appropriate *and *everyone could receive a battery-free windup radio, to listen to the radio soap-opera. But of course these potential combined gains are seldom realised. Rarely can one afford to invest fully in all programme options, everywhere, at the same time. It is also wasteful to do so, since some things only work when other things have happened first.

Level 4 indicators are the modelled investment options, asking "what if" questions. This highest level indicator re-grounds the model in a reality of costs (how much money is available to buy radios) and in possible failure rates (for example, what if only 50% of the trainers give up training about technical specifics of land mines and embrace "mine-smartness" as an approach).

Part of the challenge of introducing maps into epidemiology is their standardisation for consistent interpretation. After considerable field testing, CIET settled on three colour gradients that show up adequately with grey scales, where colour printers are not available. For level zero indicators, coverage is portrayed by shades of green (Figure 4); the level of an outcome indicator, like behaviour, is reflected by shades of yellow through brown. Level 1 indicators, reflecting relative risk, are shown by shades of blue. CIET colour schemes rely on the principle that darker shades refer to areas in need of attention or investment.

### "Morphing" the maps

An interesting output of epidemiological geomatics is the sequence of predictions based on a sample of local impacts. One might begin with a baseline occurrence, for example, the place-specific land mine incident rates, or the rate of mine-smartness (conditioned risk-avoidance behaviour). One might overlay this with a map of place-specific impacts – essentially a risk difference – after taking account of the possible confounders. Each of these maps is generated in the GIS software (initially Idrisi, then CIETmap). The resulting prediction scenario shows the revised population weighted rates of the occurrence in question. Flipping between the scenarios allows the planner to visualise the likely shift in the occurrence rate with a given investment strategy. Alternative strategies can be modelled, and the predicted effects compared.

## Conclusion: mapping is not enough

The Afghanistan evaluation produced evidence that the BBC soap opera worked while the mine training programme was in serious need of revision. The only way the programme managers could know this, was being prepared to ask the tough question. The evaluation revealed that mine risk education programmes in Afghanistan underachieved substantially, despite possibly being well run. The traditional direct training curriculum and educational delivery approaches, and the supervisory approach to project management, fell short of the challenge. By the same token, there were measurable local successes to build upon, and best practice cases that could be held up as models to be reproduced elsewhere.

The evaluation has already had a positive effect in Afghanistan. The focus of mine risk education training has shifted from technical description of mines to behaviour around mines, direct training programmes have been overhauled, and the concept of a mine committee has been reassessed. The next step is to measure and to map the improvements produced by all these changes. As an ongoing decision-making tool, a place-based approach to planning mine action could facilitate evolution of the Afghanistan programme, helping it respond in a more agile way to accommodate the continuous changes in the mine/UXO situation in the country – and the community responses to these changes.

The epidemiological geomatics exercise in Afghanistan was not an unqualified success. The factors influencing the success of a place-based approach in other settings [12] were also at work here. For example, the unifying authority has to be able to recognise and to deal with the occurrence relations in identified *types *of places. Use of UN regions of humanitarian assistance to stratify the sample should have supported incorporation of the maps in the planning by the contracting agency – but the UN involvement in mine action was limited, despite its self-proposed coordination role. An emerging lesson is that the geographic domain of the maps must coincide with the domain of the unifying authority.

Maps can be useful in demonstrating and promoting a common stake holding, but their ultimate value depends on the genuineness of the development process. Subsequent developments (and reversals) in Afghanistan indicate show the relative unimportance of planning, with or without epidemiological geomatics, in this turbulent setting.

Despite these failings, we were able to fulfil the epidemiological imperative to take account of other possible explanations of association before beginning causal inferences or making predictions based on the evidence. The maps were based on high quality epidemiological evidence, meaning data accuracy, resolution (the level of detail it offers) and currency (the usefulness in getting the job done). The sampling process allows one, within the limits of scientific probity, to generalise the findings of a sample survey *beyond the sample *to the rest of the population. Our ability to hook the last-stage random sample of the population to the exact spatial location of the cluster gives a useful coherence to the epidemiological models.

The case study illustrates the feasibility of epidemiological geomatics. Under better conditions, more reliable spatial data should be available, they should be more easily accessed and analytical skill levels may be higher. Hopefully the capacity of the contracting agencies to absorb the results might also be higher in other settings.

There is nothing new about the idea that, just as a sample can produce an interpretable model with summary predictive statistic, a sample can also be portrayed on a map as a spatial model of what happens and in what type of place it happens. The episode of the famous Broad Street pump and London's cholera epidemic probably had more to do with the careful mapping of cases and inferences from the spatial distribution of cases about the source, than it did with a seemingly definitive removal of a pump handle.

In more recent years, we have become quite accustomed to seeing maps as presentation and planning devices. A well-known example linked to prediction is the weather raster map, where colourful bands spread across a recognisable geographic domain. These rasters are models based on a sample of empirical observations, now made more comprehensive with satellite technology, projected forward in time in an attempt to predict the weather. With population weighted raster maps we can include epidemiological analysis in the presentation, allowing planners to visualise specific models of affected populations.

Population weighted raster maps overcome many of the challenges of using maps in epidemiology – and epidemiology in maps. While vector maps rely on polygon boundaries to represent data, raster maps are a grid of cells representing data independently from a usually arbitrary boundary (like a district or region). Raster maps can be used with a representative sample and the information can be interpolated from the sample locations to create a surface of an indicator on the map. The sample allows for larger amounts of specific data to be collected and followed through time at relatively low cost. The inclusion of population weights (affecting how much influence each sample location has upon the overall picture) allows the resulting maps to represent populations, rather than area, an important consideration for planners. Since the interpolation process is independent from arbitrary boundaries the data on the maps is continuous in nature – a trend which is more consistent with most population health patterns.

The complexity of calculations behind the maps does not make up for the quality of the original data. Instead of taking meta-data (where and how the evidence was collected) for granted, an expert layer can to weight the confidence in a given layer of data.

Epidemiological geomatics offer a powerful communication and mobilisation tool. Its power comes from the visual summary of (i) the population weighting of the detailed place-specific data in a scientifically coherent sample; (ii) higher level indicators that move beyond simple description to occurrence relations and (iii) the epidemiological discipline of ruling out other explanations in causality. Instead of simply reflecting the *locations*, epidemiological geomatics maps the occurrence relations or circumstances for changing risk and resilience. This is what planners need to know even when they may not have the full training to understand all the science that goes on behind the maps in a predictive sequence.

Until recently, a trained GIS technician was required even to produce simple maps. Linking of maps with epidemiological analysis was beyond the reach of most development agencies. With the advent of user-friendly epidemiological geomatics software, the situation now is reminiscent of the field of statistics a few decades ago, when the service of a statistician was the *sine qua non *of almost all research. Since then, incorporation of statistics courses in most university careers has transformed the discipline of statistics. Perhaps the same will be true of epidemiological geomatics, as planners begin to demand it to help them to address their most pressing needs. We hope that population weighted raster maps will serve as a useful tool for evolution of the field of epidemiological geomatics.

## Authors' contributions

NA developed the concepts of population weighted raster maps and designed CIETmap, the software able to implement this. He is author of the CIETmethods, and designed and oversaw implementation of the Afghanistan Mine Awareness Evaluation.

SM implemented the maps initially with Idrisi and then with CIETmap. He coauthored the technical specifications of CIETmap and oversaw the production of the software. Additional information is available on this from .
